# Effectiveness of respectful care policies for women using routine intrapartum services: a systematic review

**DOI:** 10.1186/s12978-018-0466-y

**Published:** 2018-02-06

**Authors:** Soo Downe, Theresa A. Lawrie, Kenny Finlayson, Olufemi T. Oladapo

**Affiliations:** 1 Brook Building, University of Central Lancashire, Preston, UK; 2 UNDP/UNFPA/UNICEF/WHO/World Bank Special Programme of Research, Development and Research Training in Human Reproduction (HRP), Department of Reproductive Health and Research, World Health Organization, 20 Avenue Appia, 1211 Geneva, Switzerland

**Keywords:** Respectful care, Respectful maternity care, Disrespect and abuse, Review, Africa

## Abstract

**Background:**

Several studies have identified how mistreatment during labour and childbirth can act as a barrier to the use of health facilities. Despite general agreement that respectful maternity care (RMC) is a fundamental human right, and an important component of quality intrapartum care that every pregnant woman should receive, the effectiveness of proposed policies remains uncertain. We performed a systematic review to assess the effectiveness of introducing RMC policies into health facilities providing intrapartum services.

**Methods:**

We included randomized and non-randomized controlled studies evaluating the effectiveness of introducing RMC policies into health facilities. We searched PubMed, CINAHL, LILACS, AJOL, WHO RHL, and Popline, along with ongoing trials registers (ISRCT register, ICTRP register), and the White Ribbon Respectful Maternity Care Repository. Included studies were assessed for risk of bias. Certainty of evidence was assessed using GRADE criteria.

**Findings:**

Five studies were included. All were undertaken in Africa (Kenya, Tanzania, Sudan, South Africa), and involved a range of components. Two were cluster RCTs, and three were before/after studies. In total, over 8000 women were included at baseline and over 7500 at the endpoints. Moderate certainty evidence suggested that RMC interventions increases women’s experiences of respectful care (one cRCT, approx. 3000 participants; adjusted odds ratio (aOR) 3.44, 95% CI 2.45–4.84); two observational studies also reported positive changes. Reports of good quality care increased. Experiences of disrespectful or abusive care, and, specifically, physical abuse, were reduced. Low certainty evidence indicated fewer accounts of non-dignified care, lack of privacy, verbal abuse, neglect and abandonment with RMC interventions, but no difference in satisfaction rates. Other than low certainty evidence of reduced episiotomy rates, there were no data on the pre-specified clinical outcomes.

**Conclusion:**

Multi-component RMC policies appear to reduce women’s overall experiences of disrespect and abuse, and some components of this experience. However, the sustainability of the demonstrated effect over time is unclear, and the elements of the programmes that have most effect have not been examined. While the tested RMC policies show promising results, there is a need for rigorous research to refine the optimum approach to deliver and achieve RMC in all settings.

## Plain English summary

There have been increasing reports that some women who use health facilities for childbirth experience disrespectful or even abusive care. This may be due to an increase in incidence, or to an increase in recognition. Policies to increase respectful maternity care (RMC) may, in theory, improve women’s experiences, and health outcomes, but this has not yet been demonstrated in formal studies. We looked for research studies that compared the introduction of RMC policies with the usual way of doing maternity care in that setting. We included papers in any language, and any year, listed in six databases, as well as looking at registers of on-going studies. Five studies met our inclusion criteria.

All took place in Africa. All were published between 2007 and 2017. Two were cluster-randomized trials, and three were before/after studies.

Using criteria to assess our certainty in the results of each study (‘GRADE’ criteria), we were moderately certain about findings that the policies increased women’s experiences of good quality care and of respectful care, and that they reduced physical abuse and disrespectful or abusive behaviours by staff. Evidence of reductions in non-dignified care, lack of privacy, verbal abuse, neglect and abandonment was of low certainty. There did not appear to be any difference in reports of satisfaction, but this finding was also of low certainty. The only clinical outcome reported was episiotomy rates, which were reduced, but, again, this finding was of low certainty.

We concluded that policies with multiple components to increase RMC do seem to work, by reducing women’s experiences of disrespect and abuse overall, as well as reducing some components of this experience. Policies and further research to increase RMC in different settings are recommended.

## Background

The requirement to uphold, protect, and fulfill human rights obligations at all levels of government, society, and health and maternity care systems is clear [1]. Despite this, there have been rising levels of reports of disrespect and abuse in maternity care, and, specifically, in institutional settings [2–5]. This may be due to an increase in incidence, or to an increase in recognition*.* Women’s experiences of such treatment has been linked to reduced use of maternity care provision, [3, 4, 6, 7], and may lead them to discourage other women from seeking care [6]. In some countries, experiences of non-consented invasive procedures occurring routinely during birth (termed ‘obstetric’ violence, but perpetrated by a range of care providers) have resulted in legislative changes designed to marginalize and eliminate such practices [8].

It is widely acknowledged at the policy level that all women should have the right to respectful, dignified care during labour and childbirth [9]. Promoting respectful maternal care (RMC) has been recognized as an important component of strategies to improve utilization and quality of maternity care [10].

In response to awareness of the overt abuse of women and neonates in some maternity care settings, and to the obligations to protect, respect and uphold human rights, initiatives have been enacted at all levels in some settings, from the legislature, to the direct interface between childbearing women and neonates, and caregivers. As an example of how the agenda has changed over the last 10 years, the White Ribbon Alliance have shifted from a focus on maternal mortality as a clinical phenomenon that requires structural solutions (increased skilled birth attendance, institutional births, and access to emergency obstetric care, for instance), to framing policies and practices to reduce maternal deaths within a more nuanced approach, that includes a Respectful Maternity Care Charter alongside existing responses [11]. In 2014, the WHO published a statement calling for the elimination of disrespect and abuse during facility-based childbirth [12]. As a consequence of this focus, in 2016 the WHO prioritized a question for its 2018 intrapartum care guideline on the effects of polices to increase RMC [13].

Policies and initiatives in this broad area tend to be framed in three different (though sometimes overlapping) categories: ‘humanised’ care’, ‘respectful’ care, and care based on human rights. Studies of humanised care often focus on the reduction of unnecessary, unconsented interventions, and increasing midwifery care [14]. Care based on human rights principles is focused more upstream, at access issues, or equity concerns [15]. In contrast to both of these approaches, respectful maternity care is more centrally focused on staff values, attitudes, and behaviors, as a vehicle for changing disrespectful and abusive practices in encounters with women and their families [11]. Interventions can be experienced as either positive or negative, but disrespectful and/or abusive staff attitudes and behaviours are likely to lead to traumatic experiences [16].

Audits and evaluations of RMC initiatives designed to change adverse attitudes and behaviours are promising [17]. Given the growing attention on this area, and the need for effective implementation strategies, it is now very timely to establish what works in this area, and to identify the important knowledge gaps that still remain. This paper reports on the effectiveness review undertaken during the WHO’s recent intrapartum care guideline development process to assess the current state of knowledge in this area as the basis for the guideline recommendation [13]. The aim of this review was, therefore, to establish the effectiveness of the introduction of a policy of RMC versus usual care with no RMC policy for women using routine intrapartum services.

## Review methods

We conducted this review in accordance with the Preferred Reporting Items for Systematic Reviews and Meta-Analyses (PRISMA) guidelines, and followed a protocol (available from the authors).

### Types of studies

Randomized and non-randomized controlled studies evaluating the effectiveness of introducing RMC policies into health facilities were considered for inclusion. Inclusion and exclusion criteria are given in Table 1.

**Table 1:** Study inclusion and exclusion criteria

Inclusion criteria
Controlled studies in any language or conducted at any date were included when: ● The intervention was primarily or partially designed to increase respectful care/decrease D&A (overall, and/or the following components; privacy/dignity/non-consented care/verbal abuse/physical abuse/abandonment/detention) ● The intervention was designed to impact on the intrapartum period ● The participants included women using standard intrapartum care ● Studies included a control group of any kind (RCTs, case-control or case-matched, observational longitudinal pre-post)
Exclusion criteria
Uncontrolled studies and studies where the following was the primary focus were excluded, unless respectful care was explicitly stated to be the mechanism of effect for the change: ● The intervention is primarily designed to decrease specific intrapartum interventions ● Studies designed primarily to increase the right to access to, or equity in, maternity care ● Studies designed to improve one specific aspect of care (such as companionship or communication) Policies designed to increase respectful care or its components for specific sub-groups of women using intrapartum care specifically tailored to their needs

### Types of participants

The review included interventions that had been applied broadly across a service and/or local community to improve RMC for all women, rather than interventions that were specifically tailored to address the needs of particular groups of women. Although women from marginalized groups (and their families) in all settings are more likely to be subject to disrespectful and/or abusive attitudes and behaviours from staff [18], policies designed to improve RMC for all women are likely to have the greatest impact on those who are currently most exposed to adverse attitudes and behaviours.

There is a growing interest in respectful care for the newborn. This is an important area of concern, but it was not addressed in the current review, as the focus was on healthy women using facilities for intrapartum care.

### Types of interventions

Studies on interventions to increase RMC for women defined as staff attitudes and behaviours that respect women’s basic dignity, privacy, and autonomy, the right to consent, and the right not to be exposed to verbal or physical abuse, neglect, or detention, were included.

Although the review focuses on the immediate experience of respectful or disrespectful care as a result of staff attitudes and/or behaviours, the review team recognize that the factors precipitating such behaviours may include health system failures, including gender inequalities, bullying hierarchies, and lack of pay, resources, and development opportunities.

### Types of outcomes

The outcomes that were most likely to be improved by an effective RMC policy were agreed by the Steering Group, and the Guideline Development Group for the WHO intrapartum care guideline. These outcomes are set out in Table 2. This review is focused on quantitative data. A parallel review was also undertaken for the guideline reports on qualitative findings in this area [19].

**Table 2:** Review outcomes

Maternal outcomes
Birth experience (self-reported or observed), including experience of respectful care, maternal satisfaction, sense of control, rating of birth experience, and psychological health Experiences of mistreatment (self-reported or observed), including abuse, disrespect, neglect, stigma, discrimination, poor communication or other forms Mode of birth Length of labour Use of pain relief Perineal/vaginal trauma
Fetal /Neonatal outcomes
Perinatal hypoxia-ischaemia Perinatal mortality

### Search strategy

#### Databases and other sources

PubMed, CINAHL, Lilacs, AJOL, WHO RHL, and Popline were searched, along with ongoing trials registers (ISRCT register, ICTRP register), and the White Ribbon Respectful Maternity Care Repository (that collects items relevant to respectful care or disrespect and abuse from around the world, including audits, service evaluations, and formal research). We also included regular AMDD monthly RMC updates, as they were issued.

Zetoc alerts were set up for relevant journal contents pages as they were published.

References for all papers reviewed at the full text stage were backchained to ensure that no earlier relevant studies had been missed.

#### Search terms

The terminology in this subject area is not always captured in MeSH terms, and some of the databases (for example Lilacs and AJOL) require keyword searches rather than complex Boolean chains. The searches were therefore modified to take account of these constraints. The approach was to use simple search strings to generate large numbers of hits, and then to screen the hits carefully to check for inclusion.

The searches were all carried out between July 26th and 28th 2017. Table 3 provides the search terms used by database, and the resulting hits. All searches were undertaken by SD. Inclusion was agreed by SD, KF, and OTO by consensus.

**Table 3:** Search terms used by database, and hits obtained

Database	Terms	Date searched	Hits
Pubmed	((maternity[All Fields] OR “labor, obstetric”[MeSH Terms] OR (“labor”[All Fields] AND “obstetric”[All Fields]) OR “obstetric labor”[All Fields]) OR intrapartum[All Fields] OR (“delivery, obstetric”[MeSH Terms] OR (“delivery”[All Fields] AND “obstetric”[All Fields]) OR “obstetric delivery”[All Fields]) OR intranatal[All Fields]) AND ((“policy”[MeSH Terms] OR “policy”[All Fields]) OR program$[All Fields] OR (“intervention”[All Fields]) AND ($respect[All Fields] OR dignity[All Fields] OR consent[All Fields] OR priva$[All Fields] OR rights[All Fields] OR confidential[All Fields] OR equity[All Fields] OR humanis$[All Fields] OR “abuse”[All Fields]) OR violen$[All Fields]))	26th July 2017	867
Cinhal	(matern* or labour or birth or intrapartum or intranatal) AND (*respect OR digni* OR consent OR priva* OR rights OR confidential OR equity OR humanis* OR “abuse” OR violen*) AND (policy OR program* or intervention) Limiters - Peer Reviewed; Research Article; Exclude MEDLINE records; Human Search modes - Boolean/Phrase	27th July 2017	358
Lilacs	tw:(tw:((tw:(maternity OR labour OR birth OR intrapartum OR intranatal)) AND (tw:(respect OR digni* OR consent OR priva* OR rights OR confidential OR equity OR humanis* OR “abuse” OR violen*)) AND (tw:(policy OR program* OR intervention)) AND (tw:(research OR randomised OR case-control OR before-after OR trial)) AND (instance:“regional”) AND (instance:“regional”) AND (instance:“regional”)) AND (instance:“regional”)) AND (instance:“regional”) AND (db:(“LILACS”))	27th July 2017	184
AJoL	Respectful care	28th July 2017	21
RHL	Respectful care/respect	28th July 2017	0/32
Popline	respectful care; *filtered by research report*	28th July 2017	47
ISRCTN	Respectful care/disrespect	28th July 2017	7/1
ICTRP	Respect AND birth/respect AND labor/respect AND delivery	28th July 2017	36/13/92

### Quality assessment

Risk of bias assessment for each study was undertaken using the Cochrane Risk of Bias tool [20]. The grading of the certainty of evidence was agreed by consensus between two authors (SD and TAL).

### Analysis

There was considerable heterogeneity in the methods applied by available studies on this topic as they did not use standard outcomes to measure respectful care or its components. Data were therefore not pooled or summarized, but reported descriptively, using the measures as reported by the primary study authors. GRADE criteria were applied to indicate the degree of certainty in the findings for each outcome assessed [21]. The assessment was based on consensus between two authors (SD and TAL).

## Results

### Characteristics of included studies

**Fig. 1:**
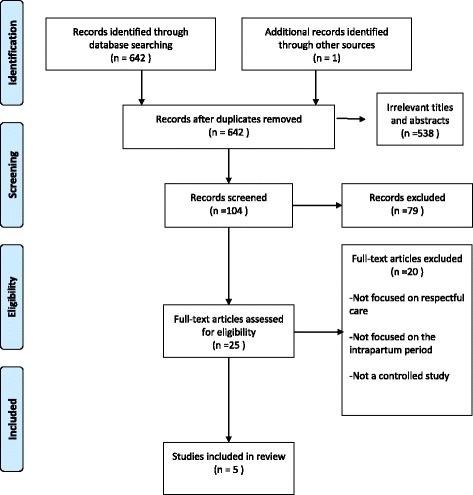
PRISMA flow chart of included studies

Of 642 hits, five studies were included (six papers) [22–27] (see Fig. 1 for the PRISMA flow chart). The characteristics of the included studies are given in Table 4.

**Table 4:** Characteristics of included studies

Study	Details
Abuya 2015, Kenya	Multicentre pre-post design Participants: Inclusion criteria at both time points: (Postbirth interviews: All women aged 15–45 who had given birth in the previous 24–48 h in the 13 Kenyan facilities taking part in the Heshima project, between Sept 2011 and Jan 2012 (baseline) and Jan and Feb 2014 (endline): Labour observations: All women aged 15–45 in labour in the included facilities/timepoints who gave consent to observation during early labour. *Interviews: N* = 641 (baseline: ‘50% of all births in the previous 48 h’) and 728 (endline: 60% of all births in the previous 48 h) *Labour observations*: *N* = 677 (baseline) and 523 (endline). It isn’t clear if some of these are the same women as those who completed surveys, or what % of those eligible agreed to take part. Intervention: Multi-component multi-system change programme designed to train maternity care providers in respectful care, as well as to reduce D&A: • Training in values and attitudes transformation • Set up of quality improvement teams • Caring for carers • D&A monitoring • Mentorship • Maternity open days • Community workshops • Mediation/alternative dispute resolution • Counseling community members who have experienced D&A Control group: (pre-intervention): Usual care (not described) *Risk of bias assessment:* lack of allocation concealment, lack of blinding, and the possible impact of interventions other than those specified in the intervention
Ratcliffe et al. 2016 (a, b) Tanzania	Single centre pre-post design Participants: *Baseline* (April-Oct 2013) For observations, ‘women presenting at the registration desk were systematically sampled’. For women’s views, every second woman on the postnatal ward at about 3–6 h postnatal systematically sampled for inclusion. 200 direct observations from admission to 2 h postpartum (paper a; reported as 208 in paper b). *N* = 2000 interviews on the postnatal ward (paper a; reported as 1914 in paper b). Subsample of 77 women re-interviewed in their homes 4–6 weeks postnatal (of the 100 who were both observed and interviewed on the postnatal ward (paper a), reported as 70 in some analyses in paper a, and as 64 in paper b. Structured interviews with all 50 local maternity providers and administrators; 18 also did in-depth interviews. *Post-intervention:* ‘Every second woman registering at the facility for delivery was selected for observation’. ‘Women who had attended an Open BirthDay (OBD) session were selected for observation’. Total observations = 459. Structured community interviews 4–6 weeks postpartum, based on ‘systematic’ selection from the OBD register and those directly observed (*n* = 149). All providers and administrators were interviewed (55/76, 72%). Intervention: a three-part step-wise dissemination and participatory process with local stakeholders from the facility, district community, and national representatives, and a multi-stakeholder working group. Two components were developed. The first (May-Oct 2014) was a series of Open Birth Days (antenatal education, communication, and information sessions for women re birth and what would happen to them in hospital, their rights, what they should bring in, open discussions between attendees and staff to build trust, tours of the hospital, including the complaints department; accompanied by posters of the ‘universal rights of childbearing women’, translated into Kiswahili and hung on all the wards, notebook copies sent to all staff, and postcard copies given to all women attending the sessions). All 362 eligible women during the intervention phase attended an OBD session. The second was a Respectful Care Workshop, held over 6 sessions over 2 days between April and May 2014, and ending with an agreed action plan agreed by each participating group, based on the WHO Health Workers for Change curriculum. 76/88 eligible staff took part, in groups of 15–20, including senior staff and administrators (in a separate group to frontline staff). Control: pre-intervention (usual care, not described) *Risk of bias assessment:* lack of allocation concealment, lack of blinding, and the possible impact of interventions other than those specified in the intervention
Kujawski et al. 2017, Tanzania	Cluster randomized study (two sites, multiple facilities in each site, approx. 60 km apart) Participants: (surveys at baseline: Dec 2011 to May 2012; and at endpoint; March to Sept 2015) Postpartum women aged 15 or over were asked to consent to take part as they left the facility. N at baseline = 1388/2085 (66.6%); n at endpoint = 1680/2324 (72.3%). Intervention (one site): 1) Participatory process with multiple community and facility stakeholders, designed to create a Client Service Charter built on consensus on norms to foster mutual respect and respectful care. The Charter was then widely disseminated in communities and local health facilities (6 months). 2) Quality Improvement process in one local facility to address D&A as a system-level issue. This comprised plan-do-act type cycles with local staff, resulting in a number of local changes such as provision of curtains to ensure privacy, transparency about stock-outs, running continuous customer satisfaction exit surveys, providing tea for on-shift staff, best-practice sharing with other wards and the regional hospital, counselling staff who showed D&A behaviours, and mutual encouragement amongst staff to exhibit respectful care (11 months managed by the research team, then 10 months without the research team, prior to formal evaluation). Control group: usual care (not described). Authors note some observed changes over time in the control site, including posting of patients’ rights in the maternity ward, a pharmacy price list, and renovations that increased service user privacy. *Risk of bias assessment:* Lack of allocation concealment, self-report outcome measures only, only one site in each arm
Umbeli et al. 2014 Sudan	Single-centre pre-post design, using structured questionnaires Participants: *Baseline:* All local health care providers (120); 10% sample of women giving birth in the hospital before the training (2000*). Post-intervention:* All local health care providers (105); 10% sample of women giving birth in the hospital after the training (2469). Intervention: Training of registrars, house officers, midwives and data collectors on communication skills, support during childbirth, providing information, and empathy. Control: pre-intervention (usual care, not described) *Risk of bias assessment:* Observational study, lack of allocation concealment, self-report outcome measures only
Brown et al. 2007, South Africa	Pilot cluster RCT (10 hospitals, randomized 5:5) Participants: sites were selected if they had more than 80 births/month from a list of maternity sites within a 200 km radius of Johannesburg. They included community, district, and referral units. Those linked to university academic departments were excluded. *Baseline:* 2090 postnatal women were interviewed from Oct 2008 (excluding those with elective CS). 2058 postnatal were interviewed in December 2009, 8 months after the intervention was introduced. Intervention; an educational intervention to promote childbirth companions to improve clinical outcomes and quality of care and promote a more woman-friendly service. Introduced to the 5 randomised sites in the two months subsequent to the introduction of the WHO RHL facilities (see below). It included an interactive workbook for use in a workshop, and the workshop itself; posters and banners encouraging women to bring in a companion; illustrated pamphlets for staff and pregnant women to show how companionship could be promoted locally; a magazine style video on birth companionship including interviews with recent South African mothers and with staff. Encouragement by the research team for senior staff to attend the workshop. The research team ran the workshop. Visits by the research team every two weeks to discuss progress, and how to overcome obstacles. Control group: usual care plus: All 10 sites were given access to the WHO Reproductive Health Library, computer hardware, and training to promote evidence based information (over two months in 1999). The 5 control hospitals were also given an evidence based intervention to promote external cephalic version, including a lecture, group discussion, a video demonstration, and an invitation to attend training in ECV *Risk of bias assessment:* Lack of allocation concealment, self-report outcome measures only

Abbreviations: D&A – disrespect and abuse; RCT – randomized controlled trial; CS – caesarean section

All the included studies were undertaken in Africa (Kenya, Tanzania (2), Sudan, South Africa). The publication date range was 2007–2017. Two were cluster RCTs (c-RCTs) [22, 23], (one with only two sites, and one with 10 sites), and three were before/after studies (four reports) [24–27]. The risk of bias assessment of all included studies revealed problems with allocation concealment, and, in most studies, with use of self-report measures alone (though this was appropriate for the outcomes that depend on women’s accounts of their views and experiences). Only one study [23] reported undertaking an a priori power calculation, though most used statistical techniques to correct findings for possible known biasing factors. Reasons for the risk of bias judgments for each study are given in Table 5.

**Table 5:** Risk of bias, GRADE assessment, and evidence profile for included studies

Population: Healthy women during childbirth Setting: Maternity, Labour wards (Tanzania, S Africa, Kenya, Sudan) Intervention: Policy to improve respectful care/reduce disrespect and abuse Control: Usual practice Data source: all data are from self-report unless specified otherwise; Publication bias could not be assessed due to few included studies									
Outcome	Quality assessment					No of participants		Relative effect A single pooled estimate is not available and only a narrative synthesis of the evidence was provided in the review	Certainty (GRADE)
	Design [studyrefs]	Risk of bias^1^	Inconsistency	Indirectness	Imprecision	RMC policy	Usual care		
Birth experience									
Respectful care	One cRCT [23] Two observational [25, 27]	Serious: cRCT 2 arms only; other data from observational studies	Not serious	Not serious	Not serious	2983 (total n for RCT) 149 and 2469 (observational)	2983 (total n for RCT) 70 and 2000 (observational)	Effect estimate for the cRCT was aOR 3.44 (2.45–4.84). Both observational studies had higher ratings of ‘respect’ in RMC arms (22.8% vs 0% in one study and 94.7% vs 89.7% in the other study)	⊕⊕⊕⊝ MODERATE Due to risk of bias
Satisfaction (very satisfied with delivery)	One cRCT [23] One observational [25]	Serious: cRCT had 2 arms only; other data were from observational study	Not serious	Not serious	Serious: wide range of effect across the two studies	2983 (total n for RCT) 149 (observational)	2983 (total n for RCT) 70 (observational)	The effect estimate for the cRCT was aOR 0.98 (0.91–1.06). The observational study showed higher satisfaction with RMC (75.8%) than control (12.9%)	⊕⊕⊝⊝ LOW Due to risk of bias and inconsistency
Good quality of care (rated good or excellent)	One cRCT [23] One observational [25]	Serious: cRCT had 2 arms only; other data were from an observational study	Not serious	Not serious	Not serious	2983 (total n for RCT) 149 (observational)	2983 (total n for RCT) 70 (observational)	The effect estimate for the cRCT was aOR 6.19 (4.29–8.94). The observational study also showed higher rating of quality of care with 63.1% vs 2.9% in RMC and control reporting this outcome	⊕⊕⊕⊝ MODERATE Due to risk of bias
Experience of mistreatment									
Any disrespectful or abusive care	One cRCT [23] Two observational [25, 26]	Serious: cRCT had 2 arms only; other data were from observational studies	Not serious	Not serious	Not serious	2983 (total n for RCT) 149 and 728 (observational)	2983 (total n for RCT) 64 and 641 (observational)	Effect estimate for the cRCT aOR 0.34 (95% CI 0.21–0.58) (3.2% vs 15.8%; RMC/ control). Observational studies had similar reductions -one from 70% to 18%; the other with aOR of 0.6 (95% CI 0.4–0.8) and rates of 13.2% vs 20.1% for RMC/control.	⊝⊕⊕⊕ MODERATE Due to risk of bias
Non-consent	Two observational [24, 26]	Serious: data were from observational studies	Serious: Direction of effect differed across the included studies	Not serious	Serious: Size of effect very different between studies	523 and 459	677 and 208	One study reported an increase [aOR 3.43 (95% CI 2.52–4.66)] with the intervention (80% vs 60.6%) and the other reported a reduction from 85.1% to 0% (all observed events)	⊕⊝⊝⊝ VERY LOW Due to risk of bias, inconsistency, and imprecision
Lack of privacy/ confidentiality	One cRCT [23] Two observational [24, 26]	Serious: cRCT had 2 arms only; other data were from observational studies	Serious: Direction of effect differed across the istudies and different measures within studies	Not serious	Serious: Effect size very different between studies and different measures	Various no.s for the different studies and measures	Various no.s for the different studies and measures	The effect estimate for the cRCT was aOR 0.25, 95% CI 0.05–1.23).The observational studies reported various measures with estimates including a range of effects between and within studies.	⊕⊝⊝⊝ VERY LOW Due to risk of bias, inconsistency, and imprecision
Physical abuse	Two cRCT [22, 23] Two observational [24, 26]	Serious: both cRCTs had methodological limitations and other data were observational	Not serious	Not serious	Not serious	2983 (total n for one cRCT) and 1039 for the other cRCT. Various n in the observational studies for different measures (according to observed or self-reported events, and different types of physical abuse	2983 (total n for one cRCT) and 1051 for the other cRCT. Various n in the observational studies for different measures (according to observed or self-reported events, and different types of physical abuse)	The effect estimate for one cRCT was aOR 0.22 (0.05–0.97). The other cRCT did not report a summary effect but had an average 50% reductionin the RMC arm (average 2% to 1%) and an increase in the control arm. Reductions in physical abuse consistently reported across the observational studies for various physical abuse measures.	⊕⊕⊕⊝ MODERATE Due to risk of bias
Verbal abuse	One cRCT [22] Two observational [24, 26]	Serious: risk cRCTs had methodological limitations and other data were observational	Not serious	Not serious	Serious: Estimates of effect include the possibility of harm	1039 for the cRCT Various n in the observational studies for different measures (according to observed or self-reported)	1051 for the cRCT 677 Various no in the observational studies for different measures (according to observed or self-reported)	cRCT did not report a summary effect, and reported little difference at follow-up in both arms. One observational study reported no clear difference (on self-report and observed measures) and the other had an absolute 49% reduction	⊕⊕⊝⊝ LOW Due to risk of bias and imprecision
Neglect/abandonment	Two cRCT [22, 23] Two observational [24, 26]	Serious: both cRCTs had methodological limitations and other data were observational	Not serious	Not serious	Serious: Estimates of effect include the possibility of harm	2983 (total n for one cRCT) and 1039 for the other cRCT; 149 and 728 for observational studies	2983 (total n for one cRCT) and 1051 for the other cRCT; 64 and 641 for observational studies	Effects differed across studies. One cRCT reported reduction with RMC [aOR 0.36 (95% CI 0.19–0.71)]. The other cRCT did not report a summary effect but reported average 33% increase in the RMC arm (from 12% to 16%). One observational study had a 38% absolute decrease. The other had no clear difference.	⊕⊕⊝⊝ LOW Due to risk of bias and imprecision
Non- dignified care	One cRCT [23] One observational [24]	Serious: cRCT had 2 arms only; other data were from an observational study	Not serious	Not serious	Serious: Estimates of effect include the possibility of harm	2983 (total n for one cRCT) 149 (observational)	2983 (total n for one cRCT) 64 (observational)	The cRCT showed no difference but direction of effect favoured reduction [aOR 0.58 (95% CI 0.30–1.12)]. The observational study showed reductions from baseline in 8/9 submeasures of non-dignified care with RMC arm (observed events); reductions in 8/9 ranged from 13.5% (mother not told where to go in AN ward) to 81.3% (provider did not introduce themselves).	⊕⊕⊝⊝ LOW Due to risk of bias and imprecision
Detention	Two observational [24, 26]	Serious: data were from observational studies	Serious: The direction of effect across the two studies differed	Not serious	Serious: Estimates of effect include the possibility of harm	149 and 728	64 and 641	One study showed an absolute decrease of 1% and the other study showed an increase [aOR 1.28 (95% CI 0.93–1.76)]	⊕⊝⊝⊝ VERY LOW Due to risk of bias, inconsistencyand imprecision
Clinical outcomes									
Perineal/ vaginal trauma	One cRCT [22]	Serious: data from observational study	Not serious	Not serious	Serious: only one study	1039	1051	This study showed a reduction in episiotomy at follow up (mean rate of 21% at RMC sites vs 39% at control sites; *P* = 0.02)	⊕⊕⊝⊝ LOW Due to risk of bias and inconsistency

High: This research provides a very good indication of the likely effect. The likelihood that the effect will be substantially different^†^ is low

Moderate: This research provides a good indication of the likely effect. The likelihood that the effect will be substantially different^†^ is moderate

Low: This research provides some indication of the likely effect. However, the likelihood that it will be substantially different^†^ is high

Very low: This research does not provide a reliable indication of the likely effect. The likelihood that the effect will be substantially different^†^ is very high

Notes on grading of Risk of Bias

All of the observational studies were assessed as having ‘serious risk’ of bias, due to lack of allocation concealment and blinding, lack of randomization, and use of self-report measures for some or all outcomes. Both cluster-RCTs were also assessed as having ‘serious risk’ of bias, due to lack of allocation concealment and blinding, and use of self-report measures for some outcomes. The RCTs were only downgraded one level for these issues as the nature of the intervention usually involved staff and/or service users in active participation, so they could not be blinded to allocation, and self-report is the only way of assessing if women experienced their care as respectful or not. Blinding of data collectors/analysts was not discussed in any of the included studies

^1^Assessment of risk of bias: All of the observational studies were assessed as having ‘serious risk’ of bias, due to lack of allocation concealment and blinding, lack of randomization, and use of self-report measures for some or all outcomes. Both cluster-RCTs were also assessed as having ‘serious risk’ of bias, due to lack of allocation concealment and blinding, and use of self-report measures for some outcomes

The types of components included in the intervention packages were highly heterogeneous. They included training in values and attitudes transformation; communication skills training; setting up quality improvement teams; disrespect and abuse monitoring; staff mentorship; improving privacy in wards (for example, with curtains or partitions between beds); improving staff conditions (for example, by providing tea for those on-shift); maternity open days; community workshops; mediation/alternative dispute resolution; counseling community members who have experienced disrespect and abuse; making provision for complaints; and educating women on their rights. One intervention was focused on companionship in labour, with an emphasis on empathic, respectful care [22], and one was focused on a communication-building package with staff [27]. The characteristics of usual care were not reported in any of the studies.

Timing of data collection ranged from direct observations of events as they happened, to follow up interviews with women at around 6 weeks after their births.

Study participants in all included studies were women utilizing facilities for intrapartum care. The total number of women involved across all included studies was around 8000 at baseline, and around 7500 at follow up (these were usually not the same women). In terms of staff, only two studies included data [24, 27], resulting in a total of 170 interviews at baseline, and 160 at follow up. Some of these were likely to be the same respondents at both time points.

All the studies reported on aspects of disrespectful or respectful maternity care. In all studies, this included women’s self-report; however, in two studies [24, 26] data were also obtained from researcher observation of practice. Apart from findings on rates of episiotomy from one paper [22] none of the other studies assessed the pre-stated clinical outcomes. Data from the included studies were not pooled due to differences in the study design and in how the outcomes were defined and reported across studies. Because data on most outcomes were relatively sparse and all of the studies were at unclear or high risk of bias, the certainty of findings was downgraded.

### Findings

The findings are reported in three broad groups: Positive birth experiences, experience of mistreatment, and clinical outcomes.

#### Positive birth experiences

We found moderate certainty evidence that implementation of a policy of RMC is more likely to result in women reporting that *the care they received was respectful* (one cRCT, approx. 3000 participants (exact numbers not stated); adjusted odds ratio (aOR) 3.44, 95% CI 2.45–4.84) [23]; two observational studies, one reporting a change from 0% to 22.8% ‘excellent’ respectful care, and the other from 89.7% to 94.7% of women reporting experiences of respectful care in the post- and pre-intervention groups, respectively [25, 27].

This was reflected in accounts of *good quality care* overall (one cRCT, approx. 3000 participants; aOR 6.19, 95% CI 4.29–8.94) [23], and in observational studies, but not in the proportion of women reporting being *very satisfied with care*, though this latter finding is based on low certainty evidence (one cRCT; aOR 0.98, 95% CI 0.91–1.06) [23], and one observational study (satisfaction 75.8% in the RMC group and 12.9% in the control group) [25].

#### Experience of mistreatment

One c-RCT [23] and two before/after studies (reported in three papers) [24–26] presented overall measures of any mistreatment or abuse. The resulting moderate certainty evidence from the cluster-RCT suggests that RMC probably reduces *experiences of disrespectful or abusive care* by about two-thirds (approx. 3000 participants; aOR 0.34, 95% CI 0.21–0.57). Observational data from the other two studies are consistent with the cRCT, with an estimated 40% relative reduction in any disrespectful or abusive care after the RMC policy introduction in one study (aOR of 0.6; 95% CI 0.4–0.8; absolute rates of 20.1% vs 13.2%) and an absolute 52% reduction in another, from 70% to 18%).

In terms of specific attitudes and behaviours, moderate certainty evidence from four studies (2 c-RCTs and 2 before/after studies) suggests that RMC policies probably reduce *physical abuse*. One c-RCT [22] reported a reduction in physical abuse in the intervention arm from a baseline average of 2% to 1% at follow-up and an increase in the control arm from a baseline average of 3% to 4% over the same time period (number of participants was not stated). The other c-RCT (approx. 3000 participants) reported an aOR of 0.22 (95% CI 0.05–0.97) [23]. One before/after study found that observed physical abuse reduced from 3.5% before the RMC intervention (677 participants) to 0.4% afterwards (523 participants) [26] and the other reported a reduction in observed use of fundal pressure from 3.4% before (208 participants) to 0.2% after (459 participants), and in ‘episiotomy without anesthesia’ of 4.3% before to 0% after [27].

Evidence on other components of disrespectful care were all graded low certainty, including evidence suggesting a reduction in *non-dignified care* (1 cRCT, approx. 3000 women; aOR 0.58, 95% 0.30–1.12) [23]. This cRCT evidence is supported by a before/after study that found large reductions in various aspects of non-dignified care (such as the provider not introducing herself to the mother, failure to provide a clean bed for the mother, and mother not being cleaned after birth) [24]. Findings from four studies (2 cluster-RCTs, and 2 before/after studies) suggest that RMC interventions may reduce *neglect and abandonment*: one cluster-RCT found a 64% reduction (approx. 3000 participants; aOR 0.36, 95% CI 0.19–0.71) [23], the other cluster-RCT reported an increase from 12% to 16%, while the observational studies found no clear difference.

Low-certainty evidence based on three studies (1 cluster-RCT, and 2 before/after study) was also contradictory for effects on *verbal abuse*. The estimates of effect in two studies included the possibility of increase, while the third study showed an absolute reduction of 49%. Similar uncertainty limits the findings on *lack of privacy* in one cRCT [23] and two before/after studies [24–26]. Various measures and inconsistent findings were reported, especially when the effects were measured over time.

Evidence on *non-consented care and detention* (keeping women at the facility against their will) was of very low certainty, with the two included studies showing effects in opposite directions.

#### Clinical outcomes

*Episiotomy* was reported in one small study [22] and the findings suggest that RMC interventions may reduce episiotomy by an average 13% (from 34% to 21%) in the RMC policy arm of this study and an average 1% (from 40% to 39%) in the control arm. However, this was graded as low-certainty evidence. The review found no evidence on mode of birth, length of labour, maternal use of pain relief, perinatal hypoxia-ischaemia or perinatal mortality.

## Discussion

Data from the five studies that met our inclusion criteria suggested that, with moderate certainty, multi-component RMC policies could increase women’s experiences of good quality care and of respectful care, and reduce experiences of disrespectful or abusive behaviours by staff, and of physical abuse. The evidence on reductions non-dignified care, lack of privacy, verbal abuse, neglect and abandonment, and reduction in episiotomy rates was less certain. There did not appear to be any difference in reports of satisfaction, but this finding was also graded as low certainty.

To date, there appear to be few well-designed, adequately powered controlled studies that have examined the impact of a policy for increasing RMC, either as a single component or package of measures, for women during labour and childbirth. Given that recognition of the nature and prevalence of disrespectful maternity care is relatively recent, this is probably not surprising. Our review is limited by the relatively high risk of bias in the included studies, partly due to the fact that only two were randomized trials, one of which included only two sites, and many of the pre-specified outcomes were not captured in these studies. The data also only represent one region of the world, and the interventions under comparison were highly heterogeneous. It was surprising that no studies were located from regions of the world other than Africa. It is possible that intervention studies in this area are framed differently (as, for example, ‘humanised care’). As noted above, we decided not to include interventions using this terminology, as such studies tend to be focused on reducing specific interventions, or introducing specific models of care, like midwifery led schemes. This omission could be a limitation of the study. However, over 8000 women are included at baseline, and over 7500 at the endpoints, and the findings of the RCT’s are generally reinforced by the observational data. It is uncommon to have so much data from low-resource settings, and the consistent direction of effect across most of the findings suggest that, even if the package of interventions varied, the multi-component design of the studies, targeted across whole health systems, was effective for at least some outcomes.

As in the analysis of survey data reported by Reis et al. [17], and the model of Jones et al. [28], the findings suggest that intervention programmes need to pay attention to the interconnectivity between the local community, the culture of the local institution, and the socio-cultural context in which both operate. Staff, as well as service users, can be affected by bullying, and disrespect [29]. This can include horizontal and vertical abuse at work and at home, especially in parallel with gender issues [30]. For staff to be enabled to enact respectful care, they must be in a system in which they, too, are respected, properly and promptly paid, able to do their job well, and where they have access to supplies, clean water, well-maintained equipment, and the potential for updating and development.

Interventions that take account of the needs of the local community, staff, service users, and the service are expensive, difficult to control for, and require authentic commitment and buy in from managerial staff, funders, and local politicians. Often such interventions need time to become fully integrated, but this also means that staff turnover and political changes can remove the people and resources that were originally committed to support the project. Issues of long-term sustainability require prolonged engagement of those intending to implement change. Despite these complications, the studies included in this review do suggest that simple elements in a package of interventions across dynamic systems of maternity care could increase women’s sense of respectful care, and of care quality, and could reduce incidences of disrespect and abuse (especially physical abuse, and high rates of episiotomy) on the basis of objective observation. The lack of effect on satisfaction is probably due to the fact that women’s reports of being satisfied are often highly skewed toward the positive, and depend on their prior expectations [31].

Qualitative research on respectful care suggests that multiple components are more likely to be effective [19]. Taken as a whole, the interventions used in included studies suggest the need for a shift in values, attitudes and beliefs, and for consistent messages and support across the whole system of care, from the local community, to front line care providers (doctors, midwives, nurses), senior professional and managerial staff, and administrators. Components ranged from caring for the carers (including making sure staff had access to tea and toast in recognition of their inability to leave the ward during a shift due to workload) to the hosting of Respectful Care Workshops, and provision of mentorship, and dispute resolution.

To ensure that RMC policies can be maximally effective in the future, policymakers and maternity service funders should ensure that infrastructure deficiencies leading to disrespectful care (e.g. state or absence of toilets and washing facilities, lack of privacy, overcrowded birth spaces) are addressed. However, such improvements require resources and time, depending on existing infrastructure and resources, and this should not inhibit the promotion and progressive rapid realization of respectful, dignified, woman-centered care for all women in terms of day to day service provision, within the context of a human rights-based approach to reducing maternal mortality and morbidity [32]. As the drivers and types of mistreatment and abuse will vary across settings, these rapid implementation schemes should ensure that local factors are clearly identified through communication with women and women’s groups in their setting/s. RMC interventions should then be tailored to addressing them among all stakeholders to optimize implementation.

Steps that can be taken very quickly at a strategic level include the development and integration of written, up-to-date standards and benchmarks for RMC that clearly define goals, operational plans and monitoring mechanisms policymakers, in collaboration with local services and communities. This work should be initiated and supported by local policy makers and maternity service funders and providers, with local birth activist groups, where they exist. Protocols for RMC and accountability mechanisms for redress in the event of mistreatment or violations, and of informed consent procedures, should be reviewed regularly. Mechanisms should be put in place to ensure that all women, and particularly those from disadvantaged backgrounds, are made aware of their right to respectful, dignified maternity care and of the process they need to use address complaints. This process should be simple, easily located, and culturally normative. It could, for example include service development and audit mechanisms that integrate women’s feedback, and ensures response to complaints.

Although the search strategy for this review did not explicitly include studies where respectful care policies were targeted at specific marginalized groups, RMC policies should recognize local contexts in which subgroups of women may be at particular risk of mistreatment, including those groups with special needs (e.g. poor awareness of their rights, and language difficulties), and ensure that RMC strategies increase levels of respectful care and equity for these women and families. Interest is also growing in the concept of respectful care for the newborn. Along with respectful care for family and friends of those using maternity services, this would be a valuable area for a review in future, as more intervention studies are published in these areas.

Policymakers should be aware that shifts in health system infrastructure, such as reorganization of staffing, or increasing workloads, could disrupt implementation. Any infrastructural changes need close monitoring to ensure and evaluate the feasibility and sustainability of RMC practices.

Current and new initiatives to implement respectful care should be formally evaluated, as a minimum with audit and/or service evaluation techniques, but ideally through controlled designs that can also allow for assessment of effects over time, such as stepped wedge designs, with or without internal action research cycles to take account of local conditions. New studies could also assess the best RMC indicators, in terms of validity and responsiveness in clinical settings, and optional implementation techniques in different kinds of settings. Ultimately, it would be important to assess the impact of increasing RMC on substantive maternal and perinatal birth outcomes, and on longer-term health and wellbeing.

## Conclusion

Despite the relative paucity of evidence to date, this review indicates that implementing multi-factored policies and practices to increase respectful care can be successful in low resource settings. This requires a visible, sustained, and participatory intervention process, with committed facility leadership, management support, and staff engagement. It is still unclear precisely which elements of a package of RMC implementation might be most successful, and most sustained over time. However, it is important to move ahead rapidly with implementation to ensure that the human rights of women, families and newborns are not violated. Studies that incorporate flexible but rigorous controlled designs, such as step-wedge implementation research, or, at the basic level, audit and service evaluation, should be undertaken alongside a widespread implementation process. Documentation of successful RMC programmes can then inform the development of guidelines and protocols for better quality maternity care in different settings.
